# The making of clandestinity: “strategic ignorance” in abortion practices in Latin America

**DOI:** 10.1080/14616742.2024.2335643

**Published:** 2024-04-22

**Authors:** Cordelia Freeman, Sandra Rodríguez

**Affiliations:** a Department of Geography, University of Exeter, Exeter, UK; b Independent scholar, Lima, Peru

**Keywords:** Strategic ignorance, ignorance studies, abortion, reproductive justice, Latin America

## Abstract

Abortion is a “public secret” in Latin America. It is highly restricted across most of the continent and yet millions of abortions take place every year. We use the sociological framework of “strategic ignorance” to argue that convenient not knowing, erasure, and concealment simultaneously prevent and facilitate abortions in Latin America. By drawing on interviews with people involved in abortion activism and access across the continent, we examine three sets of actors: the state, abortion providers, and individuals seeking abortions. When wielded by the state, strategic ignorance reproduces the status quo of the criminalization of abortion; however, when wielded by abortion providers and individuals seeking abortions, it creates the conditions for “clandestine” abortions to be procured without prosecution. Strategic ignorance is therefore mobilized by the powerful as well as the less powerful who are resisting state control of their fertility and reproductive lives.

## Introduction

In the vast majority of Latin American countries, abortion is illegal and stigmatized. However, abortions are highly common, with 6.5 million taking place across the region every year (Bearak et al. 2020). We set out to examine this apparent paradox and investigate what maintains this situation in which abortion is both allowed and suppressed. In this article, we explore how a range of actors strategically ignore, stay silent on, conceal, and erase information in ways that allow for abortion access in legally restrictive regimes in Latin America.

Paradoxically, abortion is a procedure that is both criminalized and shrouded in stigma procedure and also highly common (Duarte, Silva, and Pinto 2020; Roth 2020). The term “clandestine” is prefixed to “abortion” “to evoke what is hidden, what is concealed, what happens in the shadows but is not necessarily completely invisible” (Sutton 2017, 889). Clandestine abortions are not by definition unsafe, but they are more likely to be so, and it is low-income, rural, racialized groups who are most likely to experience unsafe abortions and to be prosecuted for accessing them. In most of Latin America, abortion is an “open secret” even when it is strictly prohibited, many people know how to access one (Céspedes, Rentería, and Pinto 2020; Kimball 2020). This builds on Bonnie Shepard’s (2000, 111) idea of the “‘double discourse system,’ which maintains the status quo in repressive or negligent public policies while expanding private sexual and reproductive choices behind the scenes.” This system results in a gulf between official, public discourse and what actually occurs in practice, which means that abortion is more commonplace and more accessible than state policy would make it seem. Abortion is a “public secret” and this paradox is reproduced through “strategic ignorance.”

This article draws on a range of interviews with people involved in abortion activism and access in Latin America: 32 in Peru and 16 in Mexico. We are unable to delve into all of the complexities of abortion access in both contexts. However, broadly speaking, abortion is highly restricted in Peru, as it is classed as a crime. “Therapeutic abortions” have been legal in theory since 1924 for specific circumstances, but in reality this pathway is inaccessible (Duffy, Freeman, and Rodríguez Castañeda 2023). At the time that the research was conducted, abortion in Mexico was legislated on a state-by-state basis, but in 2023 Mexico’s Supreme Court decriminalized abortion nationwide. This decriminalization does not create pathways to access, however, and formidable barriers to abortion remain. Regardless of the legal status of abortion, many thousands of abortions take place in both Peru and Mexico every year (Bearak et al. 2020; Ferrando 2006; Singer 2022). Therefore, these two countries can serve as prime examples of how abortions are accessed when the voluntary termination of pregnancy is illegal or restricted.

The interviews were conducted between January 2020 and November 2022. The participants were selected through purposive sampling and included abortion activists, providers, and people involved with organizations from the multinational to the local level, including state actors. These categories often overlapped, with people working in state jobs also identifying as activists. We attempted to interview participants from a variety of regions in each country, so Peruvian interviewees were in Lima, Ayacucho, Cajamarca, Arequipa, and Cusco, and Mexican interviewees were in Mexico City, Yucatán, Nevo León, and Baja California. This breadth of locations meant that geographical differences in law, access to services, and attitudes toward abortion could be covered. The interviews were wide ranging and unstructured, but centered around abortion law, provision, and access. The interview data were anonymized, transcribed, and manually coded using inductive coding independently by the authors and then compared.^1^ Any quotes used here were translated from the original Spanish by the authors. Ethical approval was granted by the University of Exeter.

In this article, we show how strategic ignorance maintains the current paradoxical situation in Latin America. On the one hand, it keeps abortion an illegal, clandestine act; on the other, it creates the conditions in which clandestine abortions can be sought and kept secret. Leslie Reagan (2022, 1) uses the term “triangle of interactions” in her work on abortion to refer to the relationships between the medical profession, state agents, and women. We adapt this triangle to focus on three actors: *the state*, as the government that controls abortion law and official practice; *abortion providers*, who are healthcare professionals, activists, and information providers; and *individuals*, who are the people seeking and accessing abortions. Diverse forms of strategic ignorance are wielded by state authorities and also by providers and individuals, which results in what Salwa Ismail (2006, xxxv) calls “the mutual ensnarement of rulers and ruled.” They work in tandem; providers and individuals are responding to state authorities, but this does not mean that the relation is unidirectional. In this article, we set out the seemingly dissimilar strategies of ignorance that together simultaneously prevent and facilitate abortions through an ambivalence that creates the scenario in which clandestine abortions continue to occur. We begin by providing an overview of the most relevant work on ignorance studies and strategic ignorance, and then explore how the three sets of actors – the state, abortion providers, and individuals seeking abortions – all produce strategic ignorance about abortion practices in Latin America.

## Locating strategic ignorance

Strategic ignorance is an idea developed by sociologists and other scholars and broadly lies within the sociology of ignorance. This body of scholarship emerged in response to work on the sociology of knowledge, on the grounds that it is just as important to study what we do not know as what we do (Stankiewicz 2009). Scholars have aimed to rectify how little is known about ignorance, and to acknowledge the social and political centrality of “knowing what not to know” (Taussig 1999, 2) and how powerful the consequences of ignorance can be (Proctor 2008).

A key aim of ignorance studies has been to show that ignorance is not a passive state; it is not neutral, natural, or simply an absence of knowledge; on the contrary, ignorance is just as socially constructed as knowledge (Reser and Smithson 1988; Sanabria 2016). This relationship between knowledge and ignorance has been extensively debated. Linsey McGoey (2012a) has challenged the assumption that ignorance and knowledge are binary opposites; she has also argued that ignorance is not the *absence* of knowledge but a *form* of knowledge (McGoey 2012b). In a similar vein, Sheldon Ungar (2008, 303) has stated that “ignorance is inextricably tethered to knowledge,” with no clear division between them, and Jutta Bakonyi (2018) has demonstrated how knowledge and ignorance can be intertwined. Furthermore, McGoey (2020) has emphasized the importance of understanding the distinction between unknowability and ignorance. While unknowability refers to the inherent inability to know something, ignorance is more contextual and suggests a refusal or failure to know something. More than a decade ago, Andrew Abbott (2010, 174) declared that there was “a certain sociological ignorance of ignorance”; however, given the breadth and depth of this scholarship, that seems to be less the case now.

Within ignorance studies, McGoey has been influential in developing work on strategic ignorance (2007, 2012a, 2012b, 2019, 2020), which she defines as “the structural ability to exploit the unknowns in an environment in order to gain more power or resources” (McGoey 2020, 198). “Structural” here means that groups deliberately conceal knowledge, “making some societal ‘unknowns’ a collective achievement” (McGoey 2020, 198). The mobilization of ignorance for nefarious reasons has been widely studied. Terence Halliday (2018) has described ignorance as something that can be cultivated; for Piotr Stankiewicz (2009), it can be wielded for political ends; and Joanna Kempner (2020) has explained that while it is an inevitable and normal aspect of life, some types of ignorance are consciously produced for deleterious ends. McGoey (2012a) has recognized the dangerous mobilization of unknowns to command resources, deny liability, and assert control; however, she has also proposed the term “emancipative ignorance,” which refers to the deliberate use of ambiguity as a weapon of resistance (McGoey 2012b). This highlights how ignorance is often an outcome of cultural and political struggle (Schiebinger 2005, 320). Ignorance can have instrumental value (Gross and McGoey 2015), and that is for the less powerful as well as the powerful.

Empirical research on ignorance has covered a range of topics, including phone hacking (McGoey 2019), nuclear threats (Reser and Smithson 1988), and scientific research (Gaudet 2013). Within health and medicine scholarship, ignorance has been studied in relation to the obesity “epidemic” in France (Sanabria 2016), medications (McGoey 2007; 2012a), human immunodeficiency virus (HIV) (Heimer 2012), the women’s health movement (Tuana 2006), and abortion (Schiebinger 2005; 2007). In her study of abortifacients in the West Indies, Londa Schiebinger (2005) has found that while the peacock flower (used in the Caribbean by slaves and non-slaves alike to provoke abortions) itself traveled to Europe, knowledge of its abortifacient properties did not. She has ascribed this to “a kind of cultured apathy or cultivated indifference” that responded to contemporary mercantilist pro-natalist policies celebrating children as “the wealth of the nation,” patterns of patronage and trade, and moral and professional imperatives (Schiebinger 2005, 342). Knowledge of the abortifacient properties of plants was therefore slowly lost, particularly in Europe.

More recent work has furthered this scholarship on the relationship between ignorance and abortion. Jessica Marcotte (2016) has shown that women have historically achieved a level of reproductive freedom not only through the creation of a system of knowledge to regulate fertility, but also through their participation in a system of ignorance that served to “sequester” this knowledge. Marcotte has explored how women historically concealed the use of “emmenagogues” – usually plants that were used to stimulate menstruation – from men, including husbands and physicians. Sally Sheldon (2018) and Irene Maffi (2022) have conducted research on how contemporary governments, policy makers, and healthcare professionals have willfully cultivated ignorance around abortion pills in the Republic of Ireland and Tunisia, respectively. This article furthers this work by exploring strategic ignorance in abortion practices in Latin America in a triangle of interactions involving the state, abortion providers, and individuals seeking abortions. This multi-actor approach recognizes that strategic ignorance is both individual and collective and is a scalar phenomenon (McGoey 2020), consisting of “varieties of ignorance” (Abbott 2010). Through these actors, we show how, at multiple scales, the cultivation of ignorance can be advantageous as both a sword and a shield.

## The state: denying the problem

Abortion is one of the main bones of contention within the shifting configurations of reproductive governance globally (Morgan and Roberts 2012). In Latin America in particular, abortion has become a so-called battleground on which liberal and conservative struggles play out. This has resulted in a split in governments’ attitudes toward the law and official practice regarding abortion. On the one hand, there has been a wave of liberalization, as seen in Argentina and Colombia; on the other, countries such as Honduras, El Salvador, Nicaragua, and the Dominican Republic have further restricted their abortion policies, narrowing the legal exceptions for an abortion or even banning the practice without exceptions (Fernández Anderson 2020). Here we argue that these latter governments deploy forms of strategic ignorance as a central part of their governmental rationality. This not only enables them to evade responsibility for unsafe abortions, but also produces the ambiguity that allows for the reproduction of clandestinity.

In the twentieth century, Latin American presidents, who were predominantly military leaders, rewrote their countries’ abortion laws as part of a broader project of modernization and updating of national legislatures. This meant that in the first third of the twentieth century, countries such as Peru, Argentina, Uruguay, and Chile changed their laws to legalize therapeutic abortions, but only in exceptional circumstances (Necochea López 2014, 57). The result was a series of restrictive abortion laws across the continent whereby any abortions that flouted these exceptional circumstances risked severe judicial punishment.

However, this criminalization of abortion does not stop it from happening; it merely forces abortions into clandestinity. While such abortions are not by default unsafe, individuals seeking abortions are left exposed “to the mercy of the elements” (Chaneton and Vacarezza 2011, 131), and any health risks disproportionately impact marginalized and racialized communities (Singer 2020; Wurtz 2012). It is also when people access healthcare services in the case of complications that they can become exposed to judicial punishment (Salazar Vega 2019). Restrictive frameworks reflect “states of uncare,” meaning not only that care responsibilities are denied but also that those who attempt to access or facilitate abortions are actively punished (Duffy, Freeman, and Rodríguez Castañeda 2023, 610).

Yet governments choose to “turn a blind eye” to these grim realities (McGoey 2012a). In part, this response is because abortion has become a battleground for its precise ability to mobilize political action, whether anti- or pro-abortion. Politicians in Latin America are often wary of being outspoken on the “costly political stance” of abortion rights because they come under attack when they defend reproductive rights or attempt reform (Fernández Anderson 2016, 17). Abortion is all too often framed by governments as a complex issue that there is simply not enough time to deal with, among other problems. For instance, in Bolivia, Evo Morales’ Movimiento al Socialismo party put the legalization of abortion aside, claiming that there was not sufficient space to add another debate to the legislative agenda (Kimball 2020). Meanwhile in Mexico, President Andres Manuel Lopez Obrador has refused to legislate on abortion, stating that it is a decision for women, not one for him to dictate from government. Some leaders believe that they can claim neutrality by being opaque about their stance on abortion.

However, we argue that deliberately choosing not to know serves a function. Governments refuse full knowledge of the magnitude of abortion practices and maintain ignorance in order to treat abortions as an anomaly. This ignorance is anything but accidental, and feigning ignorance is itself an “expression of political will” (Halliday 2018, 937). Claiming that abortion is unknowable and ungovernable is a form of governance in itself. The sheer number of clandestine abortions that take place in most Latin American countries is an inconvenient truth for governments. To maintain their structural strategic ignorance about the violent consequences of anti-abortion policies, many Latin American states deliberately avoid collecting data on the incidence of abortions. Where abortions are clandestine and occur outside of any formal health system, gathering accurate data on their frequency is always going to be a difficult task. This is illustrated by the fact that Cuba, which has legalized abortion, is able to gather reliable data about abortion, whereas countries without legal abortion, including Peru, rely on hospital admissions or targeted surveys that grossly underestimate the abortion rate (Paxman et al. 1993).

By preventing knowledge from emerging (McGoey 2020), states blinker themselves to the magnitude of the health burdens associated with clandestine abortion procedures. This manufactured unknowability serves to shape abortion as a specific problem of governance, one that pertains to the control and prosecution of deviant and “bad” women rather than to the design of public health policies. This creates a moral cognitive disconnect (Sullivan and Tuana 2007) that reinforces stigma and social sanction, further entrenching a “prevalence paradox” in which abortion exceptionality is assumed (Kumar, Hessini, and Mitchell 2009). By choosing to remain deliberately ignorant, the state can maintain the fiction that abortion is an anomaly, which in turn justifies its criminalization. Remaining ignorant enables the state to shift responsibility onto individuals, absolving it of the obligation to change any laws or provide access to healthcare. Ignorance becomes a productive asset through which the state evades responsibility and by which judicial and legal sanction become a legitimate governmental response (Stel 2016).

Given the difficulties around monitoring abortion, does the state wish to know when an illegal abortion, constituting a crime, has taken place? Prosecution is a very real threat, ruining lives and perniciously affecting those who have had a miscarriage if there is any suspicion that it may have been provoked. However, despite the vast numbers of illegal abortions taking place every year, prosecutions remain relatively low across Latin America (Fernández Anderson 2016). As Natalie Kimball (2020) has shown in the context of Bolivia, there are several reasons for this. First, it is difficult to prove that an abortion has occurred, as the effects of a medication abortion are indistinguishable from those of a miscarriage and in order for providers to be prosecuted, they need to be caught in the act of performing an abortion or someone needs to provide a confession. Second, physicians are often reluctant to report suspected abortions to the authorities because they are sympathetic, want to avoid the additional work, or do not want to make private affairs public and risk their reputation (Necochea López 2014).

Nevertheless, anxieties over being caught, interrogated, or forced to pay bribes to the police, as well as over potentially losing one’s job and the stigma of being publicly denounced or investigated, are all part of the culture of fear created by the criminalization of abortion. There are police officers, doctors, and judges who actively seek to prosecute the procurement of abortion, and those who are targeted are disproportionately economically marginalized (Fernández Anderson 2016). In the last decade in Peru, 571 women have been charged with intentionally terminating their pregnancies, and the number of complaints filed by the public prosecutor’s office is far higher (Salazar Vega 2019). In Mexico between 2007 and 2016, 3,568 people were reported to the police on suspicion of procuring or providing an abortion (GIRE 2018). The fear that people feel when seeking an abortion is legitimate.

Given the hundreds of thousands of abortions that take place across Latin America every year, this culture of fear is clearly not preventing abortions from occurring. However, rather than being the manifestation of a policy failure, we argue that the low number of prosecutions has a double utility. On the one hand, it helps to maintain the culture of fear, where the violence of sanction is administered by the effects of state *absence* rather than its presence. The government’s *potenza* (power) (Martin 2011, 195) is manifested in its potential abandonment of those who have a clandestine abortion and all of its attendant risks. On the other hand, the low number of prosecutions maintains the illusion of the efficacy of the bureaucratic apparatus of surveillance (Dedieu, Jouzel, and Prete 2015). A higher number would demonstrate that abortion is ubiquitous and highlight the inability of the state to prevent it. By keeping the number of prosecutions low and maintaining the illusion that surveillance mechanisms are effective, the state uses this form of strategic ignorance to construct public legitimacy and ensure the survival of its bureaucratic apparatus.

A second form of strategic ignorance deployed by the state is the active production of ambiguity through the obfuscation of healthcare information and rights. In her study of Palestinian gatherings under Lebanese control, Nora Stel (2016) introduces Elizabeth Cullen Dunn and Jason Cons’ (2014, 93) notion of “sensitive spaces” instead of “spaces of exception” to engage explicitly with ambiguity and uncertainty as core features of spaces where multiple modes of power and conflicting claims to sovereign control collide. Clandestinity is also determined by a pervasive uncertainty, unpredictability, and ambiguity where individuals seeking abortions have to navigate their loss of political rights. The provision of therapeutic abortions is an arena that illustrates how ambiguity and confusion can be exploited by the state in order to evade its responsibilities. Therapeutic abortions are technically legally available in most Latin American countries for very specific reasons that tend to orbit around risk to the life of the gestating parent, fetal inviability, and rape; however, this does not mean that abortion is accessible in practice even where one of these criteria is met. The technical criteria mean that on the international stage most Latin American states are seen to be meeting expectations that abortions should be legal in specific circumstances, yet in reality these legal exceptions are very hard to access. They require a panel of doctors and/or judges, paperwork, knowledge that the exceptions exist, and time. With these exceptions placed within gestational limits, by the time someone has proven that they meet one of the criteria, it may well be too late for the procedure to be performed legally. Moreover, even if legal access to abortion is granted, it may not be possible to find a clinic that will offer the procedure or a doctor willing to perform it (Pilecco et al. 2021). The result is that even those who meet the criteria for legal abortions are often denied their right, forcing them to seek clandestine abortions.

In Latin American countries that have some provisos for legal abortion but no general right to abortion, the state creates confusion and misinformation about the legal status of abortion (Palomino et al. 2011). Those who work for the state may believe that they are protecting women and “the unborn” in a paternalistic way and that withholding information about abortion is necessary to serve the public (McGoey 2007). This belief is misplaced, as countries with strict abortion laws have higher mortality rates (Latt, Milner, and Kavanagh 2019). Therefore, abortion “is often regulated in such a way that neither its safety nor women’s unburdened access to the procedure are guaranteed” (Kimball 2020, 236).

The state wields strategic ignorance by deliberately choosing not to know about the problem of abortion and by exploiting ambiguity and confusion within spaces of clandestinity. The state’s manufactured ignorance fulfills governmental logic and practice. By rendering abortion an anomaly, the state justifies its criminalization and legitimates the efficacy of the bureaucratic apparatus. As previously argued, the state is able to shift responsibility onto individuals, absolving it of the obligation to change any laws or provide access to healthcare. This form of reproductive governance upheld by so many Latin American governments is a form of “wilful blindness” (McGoey 2019); abortions that occur beyond state regulatory systems officially do not exist (Kimball 2020). At the same time, these forms of strategic ignorance produce an ironic outcome: they render the state incapable of understanding the world that it has helped to create (Mills 2007). The unknowability of clandestine abortions is what has allowed a flourishing of alternative methods to access abortions. Hence, they paradoxically provide the conditions in which clandestine abortions can be sought and kept secret.

We now turn to the forms of strategic ignorance wielded by the less powerful – abortion providers and individuals seeking abortions – which respond to the tactics of the authorities in the cracks of these marginal and clandestine spaces.

## Abortion providers: support in the shadows

Performing an abortion typically carries a severe prison sentence across the continent. As a result, abortion providers have to maintain secrecy and privacy as a necessary protection against prosecution. As one Peruvian abortion provider put it, “one of the strategies – and it angers me to say it – has been to keep quiet.” Yet, at the same time, providers face a fundamental problem: in order to be effective in expanding access to abortion, they have to be known. Therefore, their practice has to be both publicly known *and* unknown. To maintain the paradoxical nature of their practice, providers create structural strategic ignorance around their work through two interrelated strategies: concealment and erasure. The deployment of these strategies is based on a detailed knowledge of the law and the simultaneous circulation of knowledge and ignorance around abortion practices. Providers manufacture a partial ignorance, sculpting the *chiaroscuro* of what is made visible and what remains in the shadows.

There is a wide spectrum of abortion providers across Latin America. Here we focus on those who directly provide abortions or information about abortions, whether surgical or medical (through taking medication) across three main groups. First, there are private or public sector healthcare professionals, who perform surgical or medication abortions, usually in addition to their work in family planning services or obstetrics and gynecology. Second, there are *acompañantes* (accompaniers), who support others with medication abortions in person or virtually by phone or text and who often give emotional as well as practical support. *Acompañante* praxis is based in solidarity, justice, and collective care, with *acompañantes* carefully negotiating “the assemblages that control and define abortion medications” (Belfrage 2023, 25). Third, there are information providers, who disseminate information about legal abortions, abortion rights, places to procure surgical abortions, and/or how to access and self-manage medication abortions. While there are clear differences between these groups, we discuss them together here due to the intersection between them. For example, members of the Socorristas en Red, an *acompañante* network in Argentina, explicitly work with healthcare professionals to promote empathetic and anti-discriminatory abortion care on their part (Zurbriggen, Keefe-Oates, and Gerdts 2018). In addition, there is no distinct division between healthcare professionals and activists. There are healthcare professionals who work as insider activists from within the health system, and there are activists who build networks with healthcare providers (Fernández Vázquez and Szwarc 2018). For Barbara Sutton and Nayla Luz Vacarezza (2021, 11), “radical abortion activism is not necessarily about always working against the state or completely outside of it.”

While many countries in Latin America have legislation that prohibits most abortions, there are opportunities to create space for abortions or information about them at the margins of the law (Ruibal and Fernández Anderson 2020). Laws can be interpreted in ways that allow for direct action strategies such as providing information or abortions “through a broad understanding of the health exception” (Ruibal and Fernández Anderson 2020, 705). Abortion providers work in an “alegal” gray zone while resisting state control of abortion to provide care where it has been denied by the state (Singer 2022). It is precisely the detailed knowledge of the law that enables providers and activists to manufacture *partial* ignorance; they provide and circulate information among people who may need it, while also manufacturing the state’s ignorance about their practices. This “theater of ignorance” – a careful maneuvering of transparency and opacity, closure and disclosure – allows them to construct their practice as a public secret. For Peter Burke (2023, 16), secrecy is characterized by keeping a small group “in the know” and a large group “out of the loop.” Providers dissect and layer the public sphere, carefully selecting what type of information is made visible, what is kept in the shadows, and who gets to be “in the know.” Providers make sure that anti-abortion healthcare professionals and the police are unable to know that an abortion has taken place, thereby shielding themselves and individuals seeking abortions from the law. Even when the work that abortion providers do is an “open secret,” they manufacture formal denial of their practice to ensure that prosecutors would struggle to put together a convincing prosecution against them.

In this alegal gray zone, two interrelated strategies allow providers to publicly circulate information while avoiding harm from authorities: concealment and erasure. Concealment strategies include verbal caution and the performance of professed ignorance in front of authorities. Providers must be very careful with the language that they use to tell women how to abort. In communication about abortions between abortion providers (particularly information providers) and those seeking abortions, an indirect way of providing information or a creative language of secrecy and codes is often deployed.

Misoprostol is a medication that has the secondary effect of provoking abortions and is accessible due to its official use for treating stomach ulcers. While misoprostol is most effective at ending pregnancies when taken together with another pill, mifepristone, misoprostol alone has been shown to have efficacy rates of 88–93 percent in clinical studies (Cohen et al. 2005) and has transformed access to safe abortions (Calkin and Freeman 2019; Freeman 2020). Providers may draw on other national, international, or public health legal frameworks to validate their “information provision.” For example, many activists use the World Health Organization (WHO) classification of misoprostol as an “essential medicine” to justify their work, as merely providing information is not illegal, and the global mobility of abortion pills challenges national legal frameworks (Calkin 2021). Activists’ hyper-awareness of the flexibility in the law means that they know what can and cannot be said, and so information is often given exclusively using the third person. As one *acompañante* in Peru explained,

at least in writing, we don’t give any more information than is necessary, but rather as “You can read this guide,” or “The WHO recommends such a thing.” We would never say “I recommend such a thing,” or “I tell you this,” or “You should do this” if we don’t already know them. We make it a little more indirect.

Another described the importance of being careful to use the third person because you never know if you are being covertly recorded.

A creative language of secrecy and codes enables providers to feign ignorance, as euphemisms create space for denial (Thiel 2015). Providers use a range of nicknames to avoid directly using terms such as “abortion” or “misoprostol” to avoid detection. One abortion Facebook group in Mexico requires users to confirm that they will never use the word “misoprostol” in the group if they wish to join. In a Peruvian abortion Facebook group, people use playful codewords so that an external observer could not prove that these terms meant “misoprostol.” Given recent attacks on abortion providers in Latin America (Drovetta, Freeman, and Rúa 2023), we have chosen not to include the specific terms, but they often revolve around food or Catholic imagery. This creates plausible deniability and allows discussions around abortion access to remain undetected.

Another strategy of concealment is the performance of professing ignorance in front of authorities. Providers must protect themselves from prosecution if they are to continue their abortion care, and they train themselves to know when and how to deny their practice. However, this does not end with themselves; the training of individuals seeking abortions is a key part of manufacturing ignorance on behalf of authorities. One provider who performed abortions at a clinic in Peru explained that empowering the women whom they support is an important part of protection. It is imperative that these women understand what they are doing and are on the side of the providers, so that if the clinic is raided and they are questioned by the police, they will not say that they are there for an abortion. Providers check that patients are aware of the importance of this as a condition to receive care.

The providers also protect themselves through the erasure of compromising evidence or the falsification of evidence, common strategies to produce or maintain ignorance (Proctor and Schiebinger 2008). The lack of evidence makes prosecution for practicing abortions much harder and allows providers to claim ignorance about any illegal abortions having occurred. For example, one abortion provider in Peru asks people to delete any written evidence of their conversations on their mobile phones, while others never give out their real names or meet people whom they do not already know, or use “burner phones.” In this way, genuine ignorance or plausible deniability can be created.

Abortion providers are also very careful with how they record any abortions that have been performed. For example, they use anonymized spreadsheets so that no procedures can be linked to individuals. One provider explained that during police raids all paperwork and computers have been seized, so this anonymity is of utmost importance. We feel the need to be cautious here to protect our interviewees, but there are detailed and rigorous ways of creating paperwork that provide an evidence trail for why individuals were visiting a certain clinic and why they may have required a gynecological procedure. This is particularly necessary if, during a raid, the police find a patient mid-procedure, as paperwork can provide cover for both the provider and the patient. Layers of paperwork including consent forms and medical images are kept to deliberately conceal abortions and to promote ignorance on behalf of the authorities. Carol Heimer (2012) calls this obfuscation “sequestered knowledge,” as those who do not know what they are looking for are overwhelmed with bewildering paperwork.

Those involved in providing access to abortion simultaneously wield knowledge and ignorance in order to work at the margins of the law (Bakonyi 2018). Through this, providers show how the border between knowledge and ignorance is continually negotiated (Gross and McGoey 2015). They embrace the paradoxical nature of their practice as a public secret, strategizing ways to circulate information for some and create ignorance on the part of others. They rely on their detailed knowledge of the law and prosecution tactics around abortion to maintain structural strategic ignorance about the abortions that they provide as a way to protect not only their own practices but also individuals seeking abortions.

## Individuals seeking abortions: concealing the procedure

One interviewee commented: “In Peru, [abortion] has always been illegal, so in that sense we have always had to look after the issue ourselves.” Maintaining secrecy and privacy has played a fundamental role in creating pathways to abortion access in restricted settings not only for providers and activists but also for individuals seeking abortions themselves. Unlike the former, individuals do not have to deal with the paradoxical nature of the public secret of their practice; instead, they generally keep it within their close networks or to themselves. Here we consider the strategies employed by individuals to conceal their own abortions from three groups: members of their social networks who could obstruct their access to abortion, those selling them the abortion pills, and the medical staff or authorities whom they encounter if they experience complications. Concealment is guaranteed not only by staying silent but also by mobilizing deliberate ambiguity and professed ignorance in order to create a theater of ignorance that enables individuals to overcome barriers to abortion access. Shepard (2000, 115) explains that as part of the “double discourse system,” such strategies are common and “constitute an escape valve that expands citizens’ sexual and reproductive choices.” This resonates with Marcotte’s (2016) argument that “culturally induced” ignorance has allowed women to broaden their reproductive freedoms. Through this theater of ignorance, women build invaluable epistemological camouflage in their practice of fertility control (Marcotte 2016). In the face of repressive reproductive governance, they reclaim the agency of the “reproductive subject” (Morgan and Roberts 2012). While these strategies show the emancipatory and protective potential of ignorance (McGoey 2012b; 2019), it is important to note that secrecy, privacy, and concealment also come with risks.

Individuals can experience greater anxiety and stress, and are at greater risk of suffering health complications, when seeking abortions without support (Casas and Vivaldi 2014; Dides, Fernández, and Peltier 2015; Ramos, Romero, and Aizenberg 2014). Family and friends can be a crucial source of economic and emotional support for individuals, even if they do not fully agree with the decision (Duffy, Freeman, and Rodríguez Castañeda 2023; Lafaurie et al. 2005), which can mediate the effects of abortion stigma (Kumar, Hessini, and Mitchell 2009). However, they can also be a source of anxiety, exacerbate feelings of shame and guilt, or obstruct abortion access. Individuals therefore enact “strategic ploys” (Proctor 2008, 3) to conceal their pregnancy, their search for an abortion, and the procedure itself. The story of a Peruvian woman whom we interviewed illustrates how a theater of ignorance can allow for support from some while avoiding harm from others. This woman asked for help from a close friend to carry out a self-managed medication abortion. However, when the procedure was not effective, she decided that silence was no longer an option and felt obliged to tell her partner, even though she was afraid that he might physically abuse her. With the help of her friend and a health provider from the local health center, she devised a plan whereby only a partial truth was revealed to him. When she and her partner went to the local health center to be examined, the health provider convinced them that the pregnancy had a high risk of birth defects and that termination would be the best option. The provider even shared the contact details of a private clinic that performs clandestine surgical abortions. In this way, the woman created a theater of ignorance where her pregnancy was revealed but her intentions remained concealed; constructing the abortion as a medical recommendation protected her from blame and abuse from her partner and allowed her to reclaim authority over her own decision.

When self-managing medication abortions, individuals often need to procure their own abortion pills, predominantly misoprostol in Latin America, which are available from pharmacies (usually with a prescription) or on the black market. Misoprostol was originally designed as a stomach ulcer medication, and so is accessible (even if not legally) for abortions (Freeman 2020). This “double life” of misoprostol (De Zordo 2016) means that those buying the pills from a pharmacy need to avoid raising suspicion that they are going to use them for an abortion through deliberate ambiguity or find a pharmacy that is willing to sell them for that purpose. In our research, we found that prospective buyers were able to give partial or limited information about why they were buying misoprostol. This included sending someone else who did not fit the stereotype of the “aborting woman” in to buy the pills. One *acompañante* in Mexico would buy misoprostol on behalf of younger women because at her age pharmacy staff would presume that she was post-menopausal and therefore could not be seeking an abortion. Another *acompañante* explained that “getting misoprostol for women is more difficult than for men. We have got, for example, a male friend of ours to buy us the pills.” An *acompañante* group located in an area of Peru in which it was difficult to buy misoprostol from pharmacies found that if they sent *viejitos ulcerosos* (ulcerous old men), they were more likely to be successful and also to be charged a lower price. One *acompañante* explained that pharmacy staff are suspicious of these men but cannot do anything about it because there is no proof that they are buying the pills for abortions. Hence, by exploiting deliberate ambiguity with regards to their motives, they guarantee access to misoprostol.

For this purchase of misoprostol from pharmacies to work, strategic ignorance on the part of pharmacy staff is also required. They play an important role in whether access to the pills is possible, and may need to be willing to take part in a theater of ignorance to “look the other way” when they have reason to suspect that the medication is being bought for abortions. Some pharmacy staff do not ask for a doctor’s prescription even in jurisdictions in which this is required by law, while others are willing to accept suspect prescriptions. For example, one *acompañante* explained how she had been able to buy misoprostol using the same prescription for two years even though it was clearly out of date. Pharmacy staff maintain the public secret of knowing what not to know by not openly selling misoprostol for abortions. This is a widespread practice in Latin America, with one *acompañante* explaining that.

[misoprostol] is hardly ever used for what it is intended for – ulcers. Everyone who goes to the pharmacy and asks for misoprostol is getting them to have an abortion, especially when they ask for 12 pills [the WHO recommended regimen].

This is increasingly the case as more effective, cheaper ulcer medications are preferred for the treatment of gastrointestinal issues. Notably, pharmacy staff are not always altruistically providing much-needed healthcare services in a state that denies them; they often charge grossly inflated prices of up to five times higher than when sold with a prescription. However, strategic ignorance by pharmacy staff does allow for the possibility of self-managed abortions with misoprostol.

When medication abortions are performed correctly, they are almost always safe and effective; however, complications can occur in rare cases. If someone experiences severe bleeding, they may be hemorrhaging and require urgent medical treatment. Misoprostol can be taken by dissolving the pills between the gum and the cheek, under the tongue, or vaginally, and there is no blood or urine test that can identify it. This means that the effects are indistinguishable from those of a miscarriage, unless residue from the pills is discovered in the vagina. It is not uncommon for patients to admit that they have taken misoprostol in the hope that it will aid their treatment. However, in reality, the treatment is identical, and confessing to the use of misoprostol in settings in which abortion is legally restricted may spark the chain of prosecution that could end in imprisonment. By knowing how to administer the pills (that is, not vaginally) and what to say to medical staff and the police, women are able to evade the legal consequences of potential residues.

Misoprostol users are trained by *acompañantes* and providers on what to say and how to “perform” if they do have to go to hospital because of excessive bleeding. This creates a theater of ignorance through knowing what to say when they arrive at a hospital with complications arising from a clandestine abortion and whether to act devastated at this spontaneous miscarriage or shocked to find out that they were pregnant at all. Even if hospital staff suspect that an abortion may have been provoked, they are unable to prove it and are therefore absolved responsibility for reporting a suspected abortion, just as pharmacy staff can “look the other way” if they have not been given any reason to suspect that misoprostol is being bought for its abortifacient properties. Individuals are trained by providers with “scripts” to explain that they “started bleeding out of nowhere” and what symptoms, feelings, and timescale to report. Individuals are thus prepared to invoke professed ignorance and perform the role of someone who has experienced a miscarriage and construct ignorance on the part of the medical staff who treat them. For Raúl Necochea López (2014, 77), this is “a form of power at work here, a subaltern kind of power that denies access to the ‘truth’ or at least to a confession.”

The use of strategic ignorance by individuals seeking abortions in a context in which the practice is considered illegal shows the emancipative power of ignorance (McGoey 2012b). Individuals set up a theater of ignorance, where transparency and opacity regarding their condition and motives are carefully deployed depending on the different groups that they (are forced to or choose to) encounter in ways that allow them to access abortion while avoiding harm. This play of closure and disclosure is created by staying silent, invoking professed ignorance, and exploiting deliberate ambiguity. In this theater of ignorance, truth does not necessarily need to be revealed nor denied. For instance, deliberate ambiguity is used in the purchase of misoprostol, as the older women or men buying the pills are not explicitly claiming to be doing so to treat stomach ulcers, but they are strategically creating the impression that they are. In other words, the strategic deployment of ignorance helps them to maintain the secrecy and privacy needed to navigate a landscape of clandestinity where risks for women are manifold.

However, we should not lose sight of the fact that these strategies are a necessary response to a punitive state. Abortion providers and individuals seeking abortions are too often *forced* into secrecy, clandestinity, and erasure. This is not out of choice but necessity, because of the institutions of repression that make other forms of resistance challenging (Scott 1985). The use of strategic ignorance can be understood as an outcome of political struggle that is also deployed by “the weak” – providers and individuals – in ways that do not constitute a revolution or collective defiance but are much more “ordinary weapons” (Scott 1985). Such forms of emancipative ignorance become tools of survival in contexts of reproductive governance in which people are punished for managing their own fertility and reproductive lives. The problem is that, as Stel (2016) argues, the less powerful are forced to resist within the parameters of domination, rather than dismantling their foundations. As we explored earlier, strategic ignorance is a central part of governmental rationality and practice, allowing the state to evade responsibility and maintain clandestinity. For this reason, we argue that silence, secrecy, and concealment simultaneously protect and endanger individuals and providers. Hence, it is understandable why the Peruvian provider mentioned above was angered by using silence as a strategy, because she was fully aware that while it meant that abortions could be practiced, it also exposed her and her patients to multiple risks. As Shepard (2000, 115) argues, these strategies may be necessary escape valves, “but because they are makeshift, illegal, or unofficial, neither availability, safety (in the case of services), nor protections of basic rights are guaranteed.” Hence, for women who venture on journeys to terminate their pregnancies, the use of strategic ignorance blurs the line between resistance and suicide (Mbembe 2019), as they have been previously forced by the state to live at the “edge of life” (Rodríguez 2020, 23). Moreover, the use of strategic ignorance by “the weak” allows the state to continue evading responsibility, maintaining the current context of clandestinity.

## Conclusion

State departments refusing to collect data on the abortion rate. Healthcare systems that fail to provide access to abortions under their own narrow “exceptions.” A volunteer on a phone line speaking in the third person when they merely want to comfort the caller. A gynecologist writing on their spreadsheet that their patient “had a coil fitted.” A man who goes into a pharmacy claiming to be suffering from stomach ulcers. People practicing what to say if a doctor asks them when they started bleeding. These strategies may not seem to have much in common with one another, but we argue that it is their combination that creates strategic ignorance and maintains the current context of clandestine abortion in Latin America. In this article, we have made an empirical contribution to abortion scholarship by setting out the strategies that conceal abortion and a theoretical contribution to ignorance studies through exploring how strategic ignorance is enacted both “top down” and “bottom up” in individual and collective ways.

Strategic ignorance makes the possibility of access to clandestine abortions a reality. Across the triangle of interactions that we have presented here – from the state, to abortion providers, to individuals – there is power in knowing what not to know. When wielded by the state, strategic ignorance reproduces the status quo of the criminalization of abortion; however, when wielded by abortion providers and individuals seeking abortions, it creates the conditions for abortions to be procured without prosecution. Many states choose to remain deliberately ignorant, providers work strategically behind a screen of secrecy and at the margins of the law, and individuals strategize to keep pharmacy and medical staff ignorant. Through these strategies, abortion becomes a public secret, one that is known but not always known to be known.

There are clear winners and losers in this story of ignorance. Elite political groups maintain their power and significant funding while reproductive healthcare remains restricted and clandestine abortions continue, with racialized and classed effects. If strategic ignorance is “the structural ability to exploit the unknowns in an environment in order to gain more power or resources” (McGoey 2020, 198), then it is cultivated by the powerful state as well as the less powerful providers and individuals for their own ends. The emancipative strategic ignorance performed by providers and individuals is a necessary form of resistance in contexts in which abortions are not legally accessible. Ignorance and secrecy save lives, protect livelihoods, and shield people from public shaming and prosecution. The strategies outlined here create ambiguity around abortion. They mean that abortions can be accessed, and increasingly safely, without being sanctioned by the state. At the same time, however, it is important not to exaggerate the power or organizational capabilities of the state. There is not always a highly orchestrated system of ignorance masterminded by a cabal of anti-abortion elites. Decisions to ignore information or neglect to know it may be deliberate or may be inadvertent, and the line between them is not always clear (Proctor 2008). However, while strategic ignorance is utilized by the less powerful to claim bodily autonomy, it also maintains clandestinity. This further reinforces misconceptions and misinformation about abortion, reproduces stigma, and keeps abortion as a shadowy, secret phenomenon.

Yet clandestinity is not the only option. As recent developments in Argentina, Mexico, and Colombia have shown, states that have previously refused to create legal pathways to abortion access can change. Nevertheless, legislation should be viewed skeptically, as without full decriminalization and the provision of broad abortion options for all, abortions will remain inaccessible and stigmatized. As the abortion activists introduced in this article have illustrated, safe, empathetic, and supported abortions are possible beyond the state. Rather than accepting crumbs from the state, we must fight for free and autonomous reproductive justice for all.

## Note

1 Given the nature of our data, interview transcripts are not publicly available. Please contact the authors to discuss data access further.

## References

[CIT0001] Abbott, Andrew. 2010. “Varieties of Ignorance.” *American Sociologist* 41 (2): 174–189. doi:10.1007/s12108-010-9094-x.

[CIT0002] Bakonyi, Jutta. 2018. “Seeing like Bureaucracies: Rearranging Knowledge and Ignorance in Somalia.” *International Political Sociology* 12 (3): 256–273. doi:10.1093/ips/oly010.

[CIT0003] Bearak, Jonathan, Anna Popinchalk, Bela Ganatra, Ann-Beth Moller, Özge Tunçalp, Cynthia Beavin, Lorraine Kwok, and Leontine Alkema. 2020. “Unintended Pregnancy and Abortion by Income, Region, and the Legal Status of Abortion: Estimates from a Comprehensive Model for 1990–2019.” *The Lancet Global Health* 8 (9): e1152–e1161. doi:10.1016/S2214-109X(20)30315-6. 32710833

[CIT0004] Belfrage, Madeleine. 2023. “Revolutionary Pills? Feminist Abortion, Pharmaceuticalization, and Reproductive Governance.” *International Feminist Journal of Politics* 25 (1): 6–29. doi:10.1080/14616742.2022.2154688.

[CIT0005] Burke, Peter. 2023. *Ignorance: A Global History*. New Haven, CT: Yale University Press.

[CIT0006] Calkin, Sydney. 2021. “Transnational Abortion Pill Flows and the Political Geography of Abortion in Ireland.” *Territory, Politics, Governance* 9 (2): 163–179. doi:10.1080/21622671.2019.1704854.

[CIT0007] Calkin, Sydney, and Cordelia Freeman. 2019. “Trails and Technology: Social and Cultural Geographies of Abortion Access.” *Social & Cultural Geography* 20 (9): 1325–1332. doi:10.1080/14649365.2018.1509114.

[CIT0008] Casas, Lidia, and Lieta Vivaldi. 2014. “Abortion in Chile: The Practice under a Restrictive Regime.” *Reproductive Health Matters* 22 (44): 70–81. doi:10.1016/S0968-8080(14)44811-0. 25555764

[CIT0009] Céspedes, Pamela, Edana R. Rentería, and Joseph A. Pinto. 2020. “The Hidden Impact of the Clandestine Abortion in Peru.” *Journal of Public Health Policy* 41 (2): 228–229. doi:10.1057/s41271-020-00218-1. 31992811

[CIT0010] Chaneton, July, and Nayla Vacarezza. 2011. *La intemperie y lo intempestivo: experiencias del aborto voluntario en el relato de mujeres y varones*. Buenos Aires: Marea.

[CIT0011] Cohen, Jessica, Olivia Ortiz, Silvia Elena Llaguno, Lorelei Goodyear, Deborah Billings, and Imelda Martinez. 2005. “Reaching Women with Instructions on Misoprostol Use in a Latin American Country.” *Reproductive Health Matters* 13 (26): 84–92. doi:10.1016/S0968-8080(05)26202-X. 16291489

[CIT0012] Cullen Dunn, Elizabeth, and Jason Cons. 2014. “Aleatory Sovereignty and the Rule of Sensitive Spaces.” *Antipode* 46 (1): 92–109. doi:10.1111/anti.12028.

[CIT0013] De Zordo, Silvia. 2016. “The Biomedicalisation of Illegal Abortion: The Double Life of Misoprostol in Brazil.” *História, Ciências, Saúde – Manguinhos* 23 (1): 19–36. doi:10.1590/S0104-59702016000100003. 27008072

[CIT0014] Dedieu, François, Jean-Noël Jouzel, and Giovanni Prete. 2015. “Governing by Ignoring: The Production and the Function of the Under-Reporting of Farm-Workers’ Pesticide Poisoning in French and Californian Regulations.” In *Routledge International Handbook of Ignorance Studies*, edited by Matthias Gross and Linsey McGoey, 297–307. London: Routledge.

[CIT0015] Dides, Claudia, Constanza Fernández, and Gwendoline Peltier. 2015. “Aborto en Chile: cifras y testimonios que respaldan la exigencia de la legalización del aborto por tres causales.” *Nomadías* 20: 145–187.

[CIT0016] Drovetta, Irene, Cordelia Freeman, and Agustina Rúa. 2023. “Self-Care for Abortion Activists and Providers: Lessons of Law and Risk from Argentina.” *BMJ Sexual & Reproductive Health* 49 (4): 308–309. 10.1136/bmjsrh-2023-201847. 37221011

[CIT0017] Duarte, Nanda Isele Gallas, Vera Lucia Marques da Silva, and Liana Wernersbach Pinto. 2020. “A ‘amiga que já abortou’: um olhar sobre experiências partilhadas em uma comunidade virtual.” *Ciência & Saúde Coletiva* 25: 1689–1698. doi:10.1590/1413-81232020255.33442019. 32402035

[CIT0018] Duffy, Deirdre Niamh, Cordelia Freeman, and Sandra Rodríguez Castañeda. 2023. “Beyond the State: Abortion Care Activism in Peru.” *Signs: Journal of Women in Culture and Society* 48 (3): 609–634. doi:10.1086/723296. PMC7614643 37324651

[CIT0019] Fernández Anderson, Cora. 2016. “Reproductive Inequalities: As Latin America’s Pink Tide Recedes, the Struggle for Reproductive Health Reform Continues.” *NACLA Report on the Americas* 48 (1): 15–17. doi:10.1080/10714839.2016.1170291.

[CIT0020] Fernández Anderson, Cora. 2020. *Fighting for Abortion Rights in Latin America: Social Movements, State Allies and Institutions*. London: Routledge.

[CIT0021] Fernández Vázquez, Sandra, and Lucila Szwarc. 2018. “Aborto medicamentoso: transferencias militantes y transnacionalización de saberes en Argentina y América Latina.” *RevIISE: Revista de Ciencias Sociales y Humanas* 12 (12): 163–177.

[CIT0022] Ferrando, Delicia. 2006. *El aborto clandestino en el Perú: hechos y cifras*. Lima: Centro de la Mujer Peruana Flora Tristán.

[CIT0023] Freeman, Cordelia. 2020. “Viapolitics and the Emancipatory Possibilities of Abortion Mobilities.” *Mobilities* 15 (6): 896–910. doi:10.1080/17450101.2020.1803588. 34849148 PMC7612045

[CIT0024] Gaudet, Joanne. 2013. “It Takes Two to Tango: Knowledge Mobilization and Ignorance Mobilization in Science Research and Innovation.” *Prometheus* 31 (3): 169–187. 10.1080/08109028.2013.847604.

[CIT0025] GIRE. 2018. *Maternidad o castigo: la criminalización del aborto en México*. Mexico City: GIRE. Accessed March 11, 2024. https://gire.org.mx/wp-content/uploads/2019/11/Maternidad_o_castigo.pdf.

[CIT0026] Gross, Matthias, and Linsey McGoey, eds. 2015. *Routledge International Handbook of Ignorance Studies*. London: Routledge.

[CIT0027] Halliday, Terence C. 2018. “Plausible Folk Theories: Throwing Veils of Plausibility over Zones of Ignorance in Global Governance.” *British Journal of Sociology* 6 (4): 936–961. doi:10.1111/1468-4446.12605. 30289164

[CIT0028] Heimer, Carol A. 2012. “Inert Facts and the Illusion of Knowledge: Strategic Uses of Ignorance in HIV Clinics.” *Economy and Society* 41 (1): 17–41. doi:10.1080/03085147.2011.637332.

[CIT0029] Ismail, Salwa. 2006. *Political Life in Cairo’s New Quarters: Encountering the Everyday State*. Minneapolis, MN: University of Minnesota Press.

[CIT0030] Kempner, Joanna. 2020. “Post-Truth and the Production of Ignorance.” *Sociological Forum* 35 (1): 234–240. doi:10.1111/socf.12576.

[CIT0031] Kimball, Natalie L. 2020. *An Open Secret: The History of Unwanted Pregnancy and Abortion in Modern Bolivia*. New Brunswick, NJ: Rutgers University Press.

[CIT0032] Kumar, Anuradha, Laura Hessini, and Ellen M. H. Mitchell. 2009. “Conceptualising Abortion Stigma.” *Culture, Health & Sexuality: An International Journal for Research, Intervention and Care* 11 (6): 625–639. 10.1080/13691050902842741. 19437175

[CIT0033] Lafaurie, María Mercedes, Erika Troncoso, Deborah Billings, Susana Chávez, Gloria Maira, Imelda Martínez, Margoth Mora, and Olivia Ortiz. 2005. “El aborto con medicamentos en América Latina: las experiencias de las mujeres en México, Colombia, Ecuador y Perú.” *CLACAI Digital*. Accessed March 11, 2024. https://clacaidigital.info/handle/123456789/50.

[CIT0034] Latt, Su Mon, Allison Milner, and Anne Kavanagh. 2019. “Abortion Laws Reform May Reduce Maternal Mortality: An Ecological Study in 162 Countries.” *BMC Women’s Health* 19 (1): 1–9. doi:10.1186/s12905-018-0705-y. 30611257 PMC6321671

[CIT0035] Maffi, Irene. 2022. “The Production of Ignorance about Medication Abortion in Tunisia: Between State Policies, Medical Opposition, Patriarchal Logics and Islamic Revival.” *Reproductive Biomedicine & Society Online* 14: 111–120. doi:10.1016/j.rbms.2021.11.001. 35005260 PMC8717451

[CIT0036] Marcotte, Jessica. 2016. “The Agnotology of Abortion: A History of Ignorance about Women’s Knowledge of Fertility Control.” *Outskirts: Feminisms along the Edge* 34: 1–21.

[CIT0037] Martin, Diana. 2011. “The ‘Where’ of Sovereign Power and Exception: Palestinian Life and Refugee Camps in Lebanon.” (PhD thesis). Durham University.

[CIT0038] Mbembe, Achille. 2019. *Necropolitics*. Durham, NC: Duke University Press.

[CIT0039] McGoey, Linsey. 2007. “On the Will to Ignorance in Bureaucracy.” *Economy and Society* 36 (2): 212–235. doi:10.1080/03085140701254282.

[CIT0040] McGoey, Linsey. 2012a. “The Logic of Strategic Ignorance.” *British Journal of Sociology* 63 (3): 533–576. doi:10.1111/j.1468-4446.2012.01424.x. 22950467

[CIT0041] McGoey, Linsey. 2012b. “Strategic Unknowns: Towards a Sociology of Ignorance.” *Economy and Society* 41 (1): 1–16. doi:10.1080/03085147.2011.637330.

[CIT0042] McGoey, Linsey. 2019. *The Unknowers: How Strategic Ignorance Rules the World*. London: Zed Books.

[CIT0043] McGoey, Linsey. 2020. “Micro-Ignorance and Macro-Ignorance in the Social Sciences.” *Social Research: An International Quarterly* 87 (1): 197–217. doi:10.1353/sor.2020.0014.

[CIT0044] Mills, Charles. 2007. “White Ignorance.” In *Race and Epistemologies of Ignorance*, edited by Shannon Sullivan and Nancy Tuana, 11–38. Albany, NY: SUNY Press.

[CIT0045] Morgan, Lynn M., and Elizabeth Roberts. 2012. “Reproductive Governance in Latin America.” *Anthropology & Medicine* 19 (2): 241–254. doi:10.1080/13648470.2012.675046. 22889430

[CIT0046] Necochea López, Raúl. 2014. *A History of Family Planning in Twentieth-Century Peru*. Chapel Hill, NC: University of North Carolina Press.

[CIT0047] Palomino, Nancy, Miguel Ramos Padilla, Brigitte Davey Talledo, Christian Guzman Mazuelos, Jessica Carda, and Angela M. Bayer. 2011. “The Social Constructions of Unwanted Pregnancy and Abortion in Lima, Peru.” *Global Public Health* 6 (1): S73–S89. doi:10.1080/17441692.2011.590813. 21732707

[CIT0048] Paxman, John M., Alberto Rizo, Laura Brown, and Janie Benson. 1993. “The Clandestine Epidemic: The Practice of Unsafe Abortion in Latin America.” *Studies in Family Planning* 24 (4): 205–226. doi:10.2307/2939189. 8212091

[CIT0049] Pilecco, Flávia Bulegon, Cecilia Anne McCallum, Maria da Conceição Chagas de Almeida, Flávia Jôse Oliveira Alves, Aline dos Santos Rocha, Naiá Ortelan, Lígia Gabrielli, and Greice Maria de Souza Menezes. 2021. “Abortion and the COVID-19 Pandemic: Insights for Latin America.” *Cadernos de Saúde Pública* 37 (6): 1–13. 10.1590/0102-311X00322320. 34231763

[CIT0050] Proctor, Robert. 2008. “Agnotology: A Missing Term to Describe the Cultural Production of Ignorance (and Its Study).” In *Agnotology: The Making and Unmaking of Ignorance*, edited by Robert Proctor and Londa Schiebinger, 1–36. Palo Alto, CA: Stanford University Press.

[CIT0051] Proctor, Robert, and Londa Schiebinger, eds. 2008. *Agnotology: The Making and Unmaking of Ignorance*. Palo Alto, CA: Stanford University Press.

[CIT0052] Ramos, Silvina, Mariana Romero, and Lila Aizenberg. 2014. “Women’s Experiences with the Use of Medical Abortion in a Legally Restricted Context: The Case of Argentina.” *Reproductive Health Matters* 22 (44): 4–15. doi:10.1016/S0968-8080(14)43786-8. 25702064

[CIT0053] Reagan, Leslie J. 2022. *When Abortion Was a Crime: Women, Medicine, and Law in the United States, 1867–1973*. Oakland, CA: University of California Press.

[CIT0054] Reser, Joseph P., and Michael J. Smithson. 1988. “When Ignorance Is Adaptive: Not Knowing about the Nuclear Threat.” *Knowledge in Society* 1 (4): 7–27. 10.1007/BF02737056.

[CIT0055] Rodríguez, Sandra. 2020. “The Psychopolitics of Abortion: Women’s Experiences of Abortion in Peru.” MPhil dissertation, University of Cambridge.

[CIT0056] Roth, Cassia. 2020. *A Miscarriage of Justice: Women’s Reproductive Lives and the Law in Early Twentieth-Century Brazil*. Stanford, CA: Stanford University Press.

[CIT0057] Ruibal, Alba, and Cora Fernández Anderson. 2020. “Legal Obstacles and Social Change: Strategies of the Abortion Rights Movement in Argentina.” *Politics, Groups, and Identities* 8 (4): 698–713. doi:10.1080/21565503.2018.1541418.

[CIT0058] Salazar Vega, Elizabeth. 2019. “Abortar en Perú: cuando víctima y familiares son llevados a cárcel.” Ojo Público, October 22. Accessed March 11, 2024. https://ojo-publico.com/1411/abortar-en-peru-victima-y-familiares-son-llevados-carcel.

[CIT0059] Sanabria, Emilia. 2016. “Circulating Ignorance: Complexity and Agnogenesis in the Obesity Epidemic.” *Cultural Anthropology* 31 (1): 131–158. doi:10.14506/ca31.1.07.

[CIT0060] Schiebinger, Londa. 2005. “Agnotology and Exotic Abortifacients: The Cultural Production of Ignorance in the Eighteenth-Century Atlantic World.” *Proceedings of the American Philosophical Society* 149 (3): 316–343. 17290673

[CIT0061] Schiebinger, Londa. 2007. *Plants and Empire: Colonial Bioprospecting in the Atlantic World*. Cambridge, MA: Harvard University Press.

[CIT0062] Scott, James C. 1985. *Weapons of the Weak: Everyday Forms of Peasant Resistance*. New Haven, CT: Yale University Press.

[CIT0063] Sheldon, Sally. 2018. “Empowerment and Privacy? Home Use of Abortion Pills in the Republic of Ireland.” *Signs: Journal of Women in Culture and Society* 43 (4): 823–849. doi:10.1086/696625.

[CIT0064] Shepard, Bonnie. 2000. “The ‘Double Discourse’ on Sexual and Reproductive Rights in Latin America.” *Health and Human Rights* 4 (2): 110–143. doi:10.2307/4065198. 10796972

[CIT0065] Singer, Elyse Ona. 2020. “Abortion Exile: Navigating Mexico’s Fractured Abortion Landscape.” *Culture, Health & Sexuality: An International Journal for Research, Intervention and Care* 22 (8): 855–870. 10.1080/13691058.2019.1631963. 31294647

[CIT0066] Singer, Elyse Ona. 2022. *Lawful Sins: Abortion Rights and Reproductive Governance in Mexico*. Stanford, CA: Stanford University Press.

[CIT0067] Stankiewicz, Piotr. 2009. “The Role of Risks and Uncertainties in Technological Conflicts: Three Strategies of Constructing Ignorance.” *Innovation* 22 (1): 105–124. 10.1080/13511610902770636.

[CIT0068] Stel, Nora. 2016. “The Agnotology of Eviction in South Lebanon’s Palestinian Gatherings: How Institutional Ambiguity and Deliberate Ignorance Shape Sensitive Spaces.” *Antipode* 48 (5): 1400–1419. doi:10.1111/anti.12252.

[CIT0069] Sullivan, Shannon, and Nancy Tuana, eds. 2007. *Race and Epistemologies of Ignorance*. Albany, NY: SUNY Press.

[CIT0070] Sutton, Barbara. 2017. “Zonas de clandestinidad y nuda vida: mujeres, cuerpo y aborto.” *Revista Estudos Feministas* 25 (2): 889–902. doi:10.1590/1806-9584.2017v25n2p889.

[CIT0071] Sutton, Barbara, and Nayla Luz Vacarezza. 2021. “Abortion Rights and Democracy: An Introduction.” In *Abortion and Democracy: Contentious Body Politics in Argentina, Chile, and Uruguay*, edited by Barbara Sutton and Nayla Luz Vacarezza, 1–25. London: Routledge.

[CIT0072] Taussig, Michael. 1999. *Defacement: Public Secrecy and the Labor of the Negative*. Stanford, CA: Stanford University Press.

[CIT0073] Thiel, Darren. 2015. “Criminal Ignorance.” In *Routledge International Handbook of Ignorance Studies*, edited by Matthias Gross and Linsey McGoey, 256–265. London: Routledge.

[CIT0074] Tuana, Nancy. 2006. “The Speculum of Ignorance: The Women’s Health Movement and Epistemologies of Ignorance.” *Hypatia* 21 (3): 1–19. doi:10.1111/j.1527-2001.2006.tb01110.x.17111554

[CIT0075] Ungar, Sheldon. 2008. “Ignorance as an Under-Identified Social Problem.” *British Journal of Sociology* 59 (2): 301–326. doi:10.1111/j.1468-4446.2008.00195.x. 18498597

[CIT0076] Wurtz, Heather. 2012. “Indigenous Women of Latin America: Unintended Pregnancy, Unsafe Abortion, and Reproductive Health Outcomes.” *Pimatisiwin* 10 (3): 271–282. 23772229 PMC3679907

[CIT0077] Zurbriggen, Ruth, Brianna Keefe-Oates, and Caitlin Gerdts. 2018. “Accompaniment of Second-Trimester Abortions: The Model of the Feminist Socorrista Network of Argentina.” *Contraception* 97 (2): 108–115. doi:10.1016/j.contraception.2017.07.170. 28801052

